# Location versus ID: what matters to lung-resident memory T cells?

**DOI:** 10.3389/fimmu.2024.1355910

**Published:** 2024-02-05

**Authors:** Bruna Gois Macedo, Mia Y. Masuda, Henrique Borges da Silva

**Affiliations:** Department of Immunology, Mayo Clinic, Scottsdale, AZ, United States

**Keywords:** lung, tissue-resident memory CD8(+) T cells, tissue-resident memory CD4(+) T cells, T cells, route of immunization, antigen tropism

## Abstract

Tissue-resident memory T cells (T_RM_ cells) are vital for the promotion of barrier immunity. The lung, a tissue constantly exposed to foreign pathogenic or non-pathogenic antigens, is not devoid of these cells. Lung T_RM_ cells have been considered major players in either the protection against respiratory viral infections or the pathogenesis of lung allergies. Establishment of lung T_RM_ cells rely on intrinsic and extrinsic factors. Among the extrinsic regulators of lung T_RM_ cells, the magnitude of the impact of factors such as the route of antigen entry or the antigen natural tropism for the lung is not entirely clear. In this perspective, we provide a summary of the literature covering this subject and present some preliminary results on this potential dichotomy between antigen location versus antigen type. Finally, we propose a hypothesis to synthesize the potential contributions of these two variables for lung T_RM_ cell development.

## Introduction

The lung is a respiratory organ specialized in gas exchange: within its alveoli, oxygen is extracted from the air and exchanged by carbon dioxide. A consequence of this fundamental role is the constant exposure to airborne antigens, innocuous or pathogenic. Many of these antigens elicit strong T cell responses, and understanding how those responses form is crucial to define how these immune responses can either eliminate pathogenic threats or promote unwanted responses to innocuous agents. CD4^+^ and CD8^+^ T cells are primed by antigen-presenting cells in secondary lymphoid organs, and effector cells migrate towards antigen-rich sites to perform their function (1, 2). After antigen clearance, a portion of T cells survive long-term, forming memory populations which are important to mount quick, efficient responses against secondary antigen exposure (3). Memory T cells can be divided by their migratory characteristics. Circulating memory T cells (T_CIRCM_) recirculate between blood, secondary lymphoid organs, and tissues, without taking up residency; these cells can be further subdivided into central memory (T_CM_), effector memory (T_EM_) and, in the case of CD8^+^ T cells, long-lived effector cells (LLEC) (3, 4). In contrast, resident memory T cells (T_RM_) establish long-term residency in tissues, mostly barrier tissues (5). The lung is, naturally, one of these tissues. Prior evidence strongly suggests that lung T_RM_ cells are pivotal in promoting local immune responses which can either be protective against pathogens (6, 7) or deleterious – for example, in response to allergens (8).

Since their discovery (9, 10), several studies aimed to define how T_RM_ cells form in the lung, as well as their specific function. From these studies, a few notions are relatively well-established. First, both CD4^+^ and CD8^+^ T cells can form lung T_RM_ or T_RM_-like populations, and this is true in response to infections (11, 12) and to allergens (8). Second, while CD4^+^ lung T_RM_ cells are somewhat stable over time (7, 8), CD8^+^ lung T_RM_ cells are notoriously short-lived, with a faster rate of decay if compared to CD8^+^ T_RM_ cells in other tissues (13, 14). Finally, both CD8^+^ and CD4^+^ T cells form heterogeneous lung T_RM_ populations, with distinct transcriptional and functional characteristics (7, 14, 15). There are, however, several unanswered questions. One of the most important unsolved puzzles in the biology of lung T_RM_ cells lies on the nature of the signals that educate T cells to acquire a resident memory phenotype. While much evidence points out that the routes of infection (or sensitization) are paramount in defining the magnitude of the lung T_RM_ response, other works suggest that, at least partially, the type of antigen can dictate the homeostasis of lung T_RM_ cells. In this perspective article, we will briefly review previous research, provide preliminary data, and propose a hypothesis for this outstanding question.

## *Where am I from*? How the route of antigen priming can affect lung T_RM_ cell establishment

T_RM_ cell development occurs through a series of processes where initial priming, T cell sensing of peripheral tissue-derived signals, and tissue microenvironmental factors play a role in the acquisition of a T_RM_ signature (16, 17). Because of their residency establishment inside the lung parenchyma, lung T_RM_ cells must acquire certain transcriptional and protein expression characteristics. Among these characteristics, T cells (a) downregulate molecules associated with tissue egress (e.g., CCR7, S1PR1 and S1PR5), as well as upregulate molecules associated with tissue retention (e.g., TGF-βRII, CD69 and/or CD103) (17–19), and (b) express chemokine receptors such as CXCR3, which sense CCL9 and/or CCL10 released in the lung parenchyma during local immune responses (12, 20). The need for sensing of lung-derived chemokines means that optimal alterations in the lung microenvironment are paramount for the formation of lung T_RM_ cells. These changes, such as production of CCL9 or CCL10 or of IL-33, a danger signal associated with heightened lung inflammation (21), are associated with local tissue antigen recognition. Indeed, airway infections or immunizations are very effective in the generation of lung T_RM_ cells, and persistent antigen in the lungs promote long-term survival of lung T_RM_ cells (22–26). The notion that lung initial antigen encounter is necessary for optimal lung-resident T cell responses is relatively well-established (27). In response to murine influenza, local antigen encounter is needed for CD8^+^ T_RM_ cell establishment (28), and the same is true for T_RM_ cells forming in response to Bacillus Calmette-Guerin (BCG) vaccination (23, 29).

Lung mucosal sites are often the first barrier encountered by pathogens or allergens. These sites are composed of a complex network of heterogeneous epithelial cells, peripheral nervous cells, innate and adaptive immune cells, covered by a mucous layer. Each one of these components can harness the tissue inflammation following local infections or allergen sensitization. IL-33 production by epithelial cells (21), nervous system regulation of immune responses (30), and the capture of antigens by local dendritic cells (31, 32) all play a role in the initiation and sustenance of lung T cell responses. The lung, due to its physiological role, must balance the induction of such responses with maintenance of its function of gas exchange. During viral infections, for example, the balance between pathogen clearance and immune modulation is tightly regulated by the epithelial cell-immune cell axis, and dysregulation of this balance can lead to severe tissue damage (33). Consequently, the production and release of effector molecules is likely regulated even within the lung tissue.

Due to the highly controlled immune environment in the lung, the presence of adjuvants to elucidate immune responses is widely used to enhance immunogenicity in the lung. Adjuvants (which are common components of vaccines) can increase the magnitude and durability of antiviral immunity, impacting the phenotype of recruited innate cells (34). In response to infections or airway allergen sensitization, natural adjuvants are pathogen-associated molecular patterns (PAMPs) present on viruses, bacteria, fungi, protozoans, recognized by pattern recognition receptors (PRRs) expressed by epithelial and resident immune cells. The establishment of an appropriate resistant or tolerant environment, the engagement of distinct PRR combinations, results in recruitment of immune cell types and cytokines produced (35). This, associated with a combination of cytokines, chemokines, and danger signals, offer evidence that the lung microenvironment is critical to promote lung T_RM_ cell establishment.

Studies on T_RM_ cells have focused on identification of tissue-derived signals, while understanding how priming of committed precursors in distinct secondary lymphoid organs has been less explored (36). Dendritic cells, for instance, are responsible for the imprinting of specific migratory patterns in the T cells during activation. DCs in skin-draining lymph nodes induce the preferential expression of homing molecules for entry into skin, whereas DCs in mesenteric lymph nodes elicit tropism for the small intestine (37). This indicates that T_RM_ cell preconditioning in an organ-dependent way already occurs during homeostasis, whereas imprinting for tissue-selective homing occurs during T cell priming. In addition, migratory DCs from different tissues might have varying capacities for TGF-β activation in draining lymph nodes, since preconditioning was less pronounced in mediastinal lymph nodes, even though these tissues had comparable induction of CD103 in naïve T cells at both sites (38). This adds another layer on how the route of antigen priming can regulate the quality and/or magnitude of lung T_RM_ cell establishment.

Other routes of antigen entry, such as intramuscular immunizations, can also induce lung T_RM_ cells, but the phenotype of these cells seems to be heterogenic, with lower proportion of cells located in the lung parenchyma (39). Thus, intramuscular immunizations have traditionally been considered poor inducers of mucosal T_RM_ cell responses (40, 41). Intranasal vaccination strategies can induce strong protection, as evidenced by past studies on RSV, Mtb and influenza (23, 26, 27, 42–44). However, this route has potential issues in antigen delivery to dendritic cells in the respiratory tract, perhaps due to physical barriers such as nasal clearing or mucus (45–47). A combination of intramuscular (i.e., distal antigen priming) and intranasal immunization approaches has been suggested as a candidate to enhance lung T_RM_ cell development in response to vaccines (41, 48, 49). Another evidence from vaccination also challenges the notion that lung antigen priming is the sole factor inducing optimal lung T_RM_ cells: the recent revolution in mRNA vaccines to combat SARS-CoV-2 (50) and, more recently, influenza (51). These immunizations, which are intramuscular, lead to robust lung CD4^+^ and CD8^+^ T_RM_-like cell responses, as studies in mice suggest (48, 51). Future studies will be necessary to identify how mRNA vaccines promote lung T_RM_ cells even without intranasal priming, and whether long-lived lung T_RM_ cells are generated in humans. It is important to note that, although these studies suggest that intranasal immunization is not strictly necessary for lung T_RM_ cell responses, intranasal priming is still sufficient to improve lung T_RM_ cell establishment in these cases (23, 26, 27).

## *Who am I*? How antigen nature and tropism can influence lung T_RM_ cell establishment

**Figure 1:**
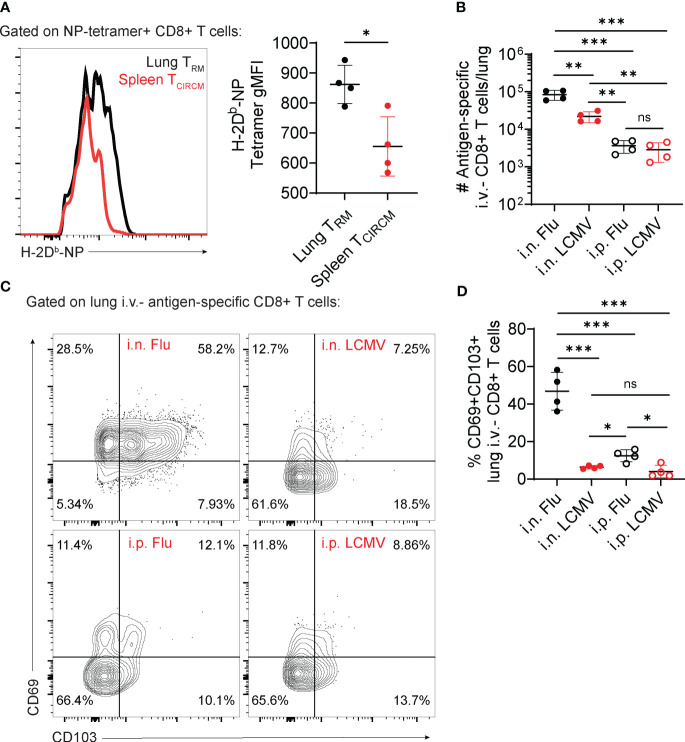
Contribution of antigen nature versus route of priming for lung CD8^+^ T_RM_ cell establishment. **(A–D)** C57BL/6 mice were infected with influenza, PR8 strain (Flu) or LCMV-Armstrong (LCMV) through the indicated infection routes (intranasal – i.n; intraperitoneal – i.p.). At day 28 post-infection, the number and phenotype of antigen-specific CD8^+^ T cells were assessed. **(A)** Representative histogram (left) and average gMFI values (right) of H-2D^b^-NP tetramer staining in NP tetramer+ lung versus spleen CD8^+^ T cells. **(B)** Average numbers of antigen-specific lung i.v.^-^ CD8^+^ T cells (CD44^+^ H-2D^b^-tetramer^+^). **(C)** Representative flow cytometry plots showing expression of CD69 and CD103 in antigen-specific lung i.v.- CD8^+^ T cells. **(D)** Average percentages of CD69^+^CD103^+^ antigen-specific lung i.v.- CD8^+^ T cells. Data from two independent experiments, n=4. **(A)** Unpaired t-test **(B, D)** One-way ANOVA with Tukey’s post-test, *p<0.05, **p<0.01, ***p<0.001. ns, not significant.

In contrast with evidence for route of antigen priming, other factors may also dictate the generation of lung T_RM_ cells, for example antigen (pathogen) load, pathogen life cycle characteristics, or the strength of TCR-MHC interaction. In mouse models of viral infection, CD8^+^ T_RM_ cells in brain and kidney express higher affinity to MHC class I tetramers (> 20x) than T_CIRCM_ cells (52). We observed a similar trend in preliminary experiments comparing influenza-specific lung T_RM_ cells with T_CIRCM_ cells (**Figure 1A**), suggesting that a selection of high-affinity clones may also happen for lung CD8^+^ T_RM_ cells.

A preferential selection of lung T_RM_ cells with high TCR affinity for antigen could either occur at the effector stage, after T cells migrated to the lung tissue, or at the priming and activation stage, in secondary lymphoid organs. Although these two hypothetical scenarios would suggest a major effect of the route of priming as a selector of lung T_RM_ cells, different lung viral infections induce distinct magnitudes of a lung T_RM_ cell response. In mice, while in response to influenza >50% of lung memory CD8^+^ T cells express the T_RM_ markers CD69 and CD103 (14), in response to RSV or BCG these numbers are much lower (23, 53). These differences may suggest that distinct antigen types or differences in TCR affinity regulate the establishment and phenotype of lung T_RM_ cells. Alternatively, they could also be explained by differences in how distinct pathogens interact with the lung immune system, for example differences in induction of cytokine production.

To test the potential contributions of route of priming versus antigen type, we infected mice with LCMV, Armstrong strain (a systemic virus with no tropism for the mouse lung) or influenza, using intraperitoneal versus intranasal infection routes. Intranasal infection with LCMV or influenza led to significantly increased numbers of lung parenchymal antigen-specific CD8^+^ T cell accumulation (**Figure 1B**). Per se, these results are indicative of the importance of airway antigen entry in the formation of lung T_RM_ cells. However, the magnitude of lung T_RM_ cell accumulation is higher in response to intranasal influenza if compared to intranasal LCMV (**Figure 1B**). A more detailed characterization of these lung T_RM_ cells also show that intranasal influenza is unique in promoting upregulation of CD69 and CD103, in comparison to intranasal LCMV (**Figures 1C, D**). Confirming previous findings (27, 54), intraperitoneal influenza, despite failing to promote the numerical accumulation of lung T_RM_ cells (**Figure 1B**), was sufficient to induce a consistent upregulation of CD69 and CD103 in a small proportion of lung T_RM_ cells (**Figures 1C, D**). These preliminary findings suggest that, despite an important role for the intranasal route of immunization, the acquisition of a classic lung T_RM_ phenotype may strongly rely on the antigen type, more specifically their natural lung tropism.

## Conclusions and a proposed hypothesis

**Figure 2:**
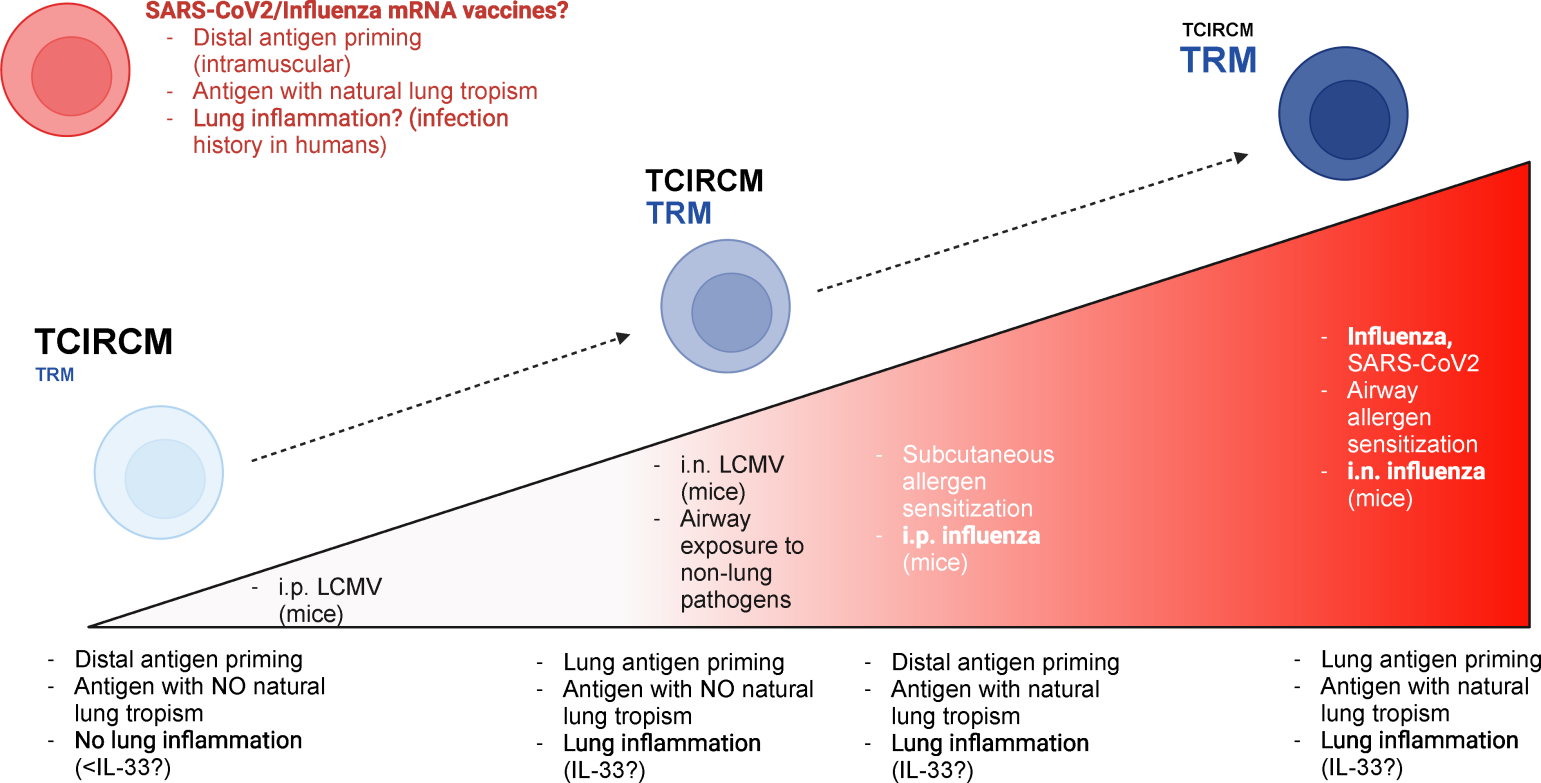
Hypothetical model for the relative contributions of antigen priming location versus antigen nature for lung T_RM_ generation. Based on past research and on our preliminary results, we postulate that the T_RM_ education of lung T cells relies on both local antigen priming and the lung tropism of relevant antigens. The magnitude of lung T_RM_ responses is at its peak when lung-tropic antigens are recognized locally (i.e., in the lung); this is the case, for example, in response to respiratory infections such as influenza or SARS-CoV-2 or to intranasal allergen sensitization. On the other hand, recruitment of lung T_RM_ cells is minimal when antigens with no lung tropism are recognized distally (e.g., systemic, or subcutaneous). When antigens with lung tropism are introduced through non-airway routes, the magnitude of lung T_RM_ cell accumulation is greatly reduced, but some accumulation may still occur; lung inflammatory responses may play an essential role in this scenario, by creating chemotactic signals for lung T cell infiltration. An example of this situation in real life is subcutaneous exposure to lung allergens. Finally, local recognition of antigens with no lung tropism, at least in experimental models, can induce a relevant accumulation of lung parenchymal T cells; however, these cells still fail to acquire a bona-fide T_RM_ phenotype, perhaps due to diminished lung inflammation – since the lack of lung tropism hinders the ability of such antigens to establish in the lung tissue. A possible exception to this rule is the product of mRNA vaccines to SARS-CoV-2 or influenza (which are in clinical trials). These immunizations, which are distal (i.e., intramuscular), would theoretically lead to intermediate lung T_RM_ responses based on our model; however, current evidence suggests a strong lung T cell accumulation upon immunization. It is possible that a heightened state of basal lung inflammation (due to past infection history in humans) could tip the balance in favor of stronger lung T_RM_ cell responses, even in the absence of local priming. This figure was generated using BioRender.

Most past studies strongly suggest that airway exposure to antigens is an important factor in the establishment of lung T_RM_ cells, but additional evidence from us and others also point to the antigen type, more specifically its natural lung tropism, as another regulating factor. We believe that optimal lung T_RM_ cell generation will take advantage of these two variables, and the magnitude of lung T_RM_ cell responses obeys a continuum (**Figure 2**). In response to airway exposure to lung allergens or to respiratory infections, both lung antigen tropism and airway route of exposure are present, and consequently a strong lung T_RM_ cell response is mounted. On the other end of the spectrum, systemic infections with pathogens lacking lung tropism do not elicit lung T_RM_ cell responses. In “hybrid” scenarios, such as intraperitoneal exposure to influenza or intranasal exposure to LCMV, lung T_RM_ cell generation will be partial, with the magnitude of the response relying on other factors induced by either lung inflammatory responses or antigen persistence.

Some questions, however, are still unanswered. First, what is the exact influence of lung inflammatory responses (such as the ones induced by innate immune cells or epithelial cells) in this “T_RM_ continuum”? When considering our intranasal LCMV system, for example, the lack of a CD69/CD103 phenotype can be due to changes during T cell priming, but the lack of lung tropism of LCMV possibly also translates in decreased infectivity in the lungs. Consequently, inflammatory responses during the acute phase are expected to be lower in lungs, which could influence the local release of signals such as TGF-β, which are necessary to educate nascent T_RM_ cells for CD103 expression (55). CD8^+^ T_RM_ cells can be generated in tissues without antigen if sterile inflammation is administered to such tissues simultaneously to systemic antigen immunization. This is true for skin T_RM_ cells (56, 57) and female reproductive tract T_RM_ cells (58). Future systematic investigations on whether such “prime and pull” strategies are sufficient for lung T_RM_ cell generation will be important.

Another important point to consider is the fact that CD4^+^ and CD8^+^ T_RM_ cells, despite sharing common pathways and molecular requirements, are not the same. CD4^+^ T_RM_ cells typically locate outside of epithelial sites, partly due to their inability to respond to TGF-β – which is controlled by their downregulation of Runx3 (59). This is also true in the lungs, where CD4^+^ T_RM_ cells are mostly concentrated within the lung parenchyma, with some of them in close contact with B cells and other immune cells (7, 15). Our model heavily takes into consideration our findings with lung CD8^+^ T_RM_ cells, as well as the abundant literature on these cells (6, 13, 14, 28). It will be interesting to assess the relative contributions of the route of priming versus antigen tropism (versus local inflammation) for lung CD4^+^ T_RM_ cell establishment. In conclusion, in this perspective we provided a short review of the known literature on how lung T_RM_ cells form, and how lung tissue versus antigen type can influence their formation. Understanding the relative roles of each one of these variables will lead, in our opinion, to the discovery of more efficient approaches to boost the generation of lung T_RM_ cells that can provide protection against infections – or to block undesirable lung T_RM_ cell formation in response to lung allergens.

## Materials and methods

### Mice

Male and female 6- to 8-week-old adult C57BL/6 (B6) mice were purchased from Jackson and were allowed to acclimate to our housing facilities for at least one week. Animals were maintained under specific-pathogen-free conditions at Mayo Clinic Arizona. In all experiments, mice were randomly assigned to experimental groups. All experimental procedures were approved by the institutional animal care and use committee at Mayo Clinic Arizona (IACUC A00005542-20).

### Viral strains

LCMV (Armstrong strain) was maintained at −80°C until infection and diluted to 2x10^6^ PFU/ml in PBS. Influenza (PR8 strain) was maintained at −80°C and diluted to 7x10^4^ PFU/ml in PBS (intranasal infection) or 7x10^7^ PFU/ml in PBS (intraperitoneal infection) at the time of infection studies.

### Infection studies

Mice were infected with LCMV-Armstrong (2x10^5^ PFU, intraperitoneally or intranasally). Other mice were infected with Influenza-PR8 (100 PFU, intranasally or 1x10^6^ PFU, intraperitoneally).

### Flow cytometry

Lymphocytes were isolated from spleen or lungs as previously described (60, 61). Lungs were removed and cut in small pieces into Erlenmeyer flasks containing 30 mL of 0.5 mg/ml Collagenase type IV. During isolation of lymphocytes from lungs, in all experiments, 50 μg of Treg-Protector (anti-ARTC2.2) nanobodies (BioLegend) were injected i.v. 30 minutes prior to mouse sacrifice (62). Direct *ex vivo* staining was performed as described (60). To identify LCMV-specific or Flu-specific CD8^+^ T cells, tetramers were obtained from the Yerkes NIH Tetramer Core: D^b^-gp33 and D^b^-NP-flu tetramers conjugated with APC- or PE-Streptavidin were used. For detection of vascular-associated lymphocytes in non-lymphoid organs, *in vivo* i.v. injection of PerCP-Cy5.5-conjugated CD8α antibody was performed (63). Among LCMV- or Flu-specific CD8^+^ T cells, the following markers were used to distinguish lung T_RM_ cells: i.v.CD8α^-^CD69^+/−^CD103^hi/int/lo^. In all flow cytometry experiments, Live/Dead Near-IR was used to distinguish between live and dead cells. Flow cytometric analyses were performed on FACS Symphony (BD Biosciences) and data was analyzed using FlowJo software (Treestar).

### Statistical analyses

Data were subjected to the Kolmogorov-Smirnov test to assess normality of samples. Statistical differences were calculated by using unpaired two-tailed Student’s t-test (or one-way ANOVA with Tukey post-test, where indicated). All experiments were analyzed using Prism 9 (GraphPad Software). Graphical data was shown as mean values with error bars indicating the SD. P values of < 0.05 (*), < 0.01 (**), < 0.001 (***) indicated significant differences between groups.

## Data availability statement

The raw data supporting the conclusions of this article will be made available by the authors, without undue reservation.

## Ethics statement

The animal study was approved by IACUC, project number A00005542-20. The study was conducted in accordance with the local legislation and institutional requirements.

## Author contributions

BM: Conceptualization, Writing – original draft, Writing – review & editing. MM: Data curation, Formal analysis, Investigation, Writing – original draft, Writing – review & editing. HB: Conceptualization, Data curation, Formal analysis, Funding acquisition, Project administration, Resources, Supervision, Writing – original draft, Writing – review & editing.
